# Spatial analysis of colorectal cancer outcomes and socioeconomic factors in Virginia

**DOI:** 10.1186/s12889-021-11875-6

**Published:** 2021-10-21

**Authors:** Esther J. Thatcher, Fabian Camacho, Roger T. Anderson, Li Li, Wendy F. Cohn, Pamela B. DeGuzman, Kathleen J. Porter, Jamie M. Zoellner

**Affiliations:** 1 University Hospitals, Cleveland, OH USA; 2 Department of Public Health Sciences, School of Medicine, University of Virginia, Charlottesville, USA; 3 Department of Family Medicine, School of Medicine, University of Virginia, Charlottesville, USA; 4 School of Nursing, University of Virginia, Charlottesville, USA

**Keywords:** Colorectal neoplasms, Incidence, Risk factors, Spatial regression, Virginia

## Abstract

**Background:**

Colorectal cancer (CRC) disparities vary by country and population group, but often have spatial features. This study of the United States state of Virginia assessed CRC outcomes, and identified demographic, socioeconomic and healthcare access contributors to CRC disparities.

**Methods:**

County- and city-level cross-sectional data for 2011–2015 CRC incidence, mortality, and mortality-incidence ratio (MIR) were analyzed for geographically determined clusters (hotspots and cold spots) and their correlates. Spatial regression examined predictors including proportion of African American (AA) residents, rural-urban status, socioeconomic (SES) index, CRC screening rate, and densities of primary care providers (PCP) and gastroenterologists. Stationarity, which assesses spatial equality, was examined with geographically weighted regression.

**Results:**

For incidence, one CRC hotspot and two cold spots were identified, including one large hotspot for MIR in southwest Virginia. In the spatial distribution of mortality, no clusters were found. Rurality and AA population were most associated with incidence. SES index, rurality, and PCP density were associated with spatial distribution of mortality. SES index and rurality were associated with MIR. Local coefficients indicated stronger associations of predictor variables in the southwestern region.

**Conclusions:**

Rurality, low SES, and racial distribution were important predictors of CRC incidence, mortality, and MIR. Regions with concentrations of one or more factors of disparities face additional hurdles to improving CRC outcomes. A large cluster of high MIR in southwest Virginia region requires further investigation to improve early cancer detection and support survivorship. Spatial analysis can identify high-disparity populations and be used to inform targeted cancer control programming.

## Background

Globally and in the United States (U.S.), colorectal cancer (CRC) is the third most common cause of cancer incidence and the second highest cause of cancer death [1–3]. Epidemiological patterns and risk factors vary within and among countries and regions of the world. Lifestyle-based risk factors such as diet and smoking vary greatly, as well as preventive measures such as screening test access and mass education campaigns [3]. Disparities in CRC refer to population subgroups that experience worse outcomes due to social, economic, other factors that impede their access to preventive services or to practice healthy lifestyles [4]. Though the characteristics and risk factors of groups experiencing CRC disparities can vary by place, studying and measuring disparities is important for informing efforts to improve health equity.

In the U.S., disparities in CRC incidence and mortality are associated with population characteristics, such as racial and ethnic minority status, low socioeconomic status (SES), rural residence, and lack of access to healthcare [5, 6]. African Americans (AA) have higher rates of cancer-related mortality than whites; this gap has grown even with mortality rates decreasing overall [7]. Populations residing in low SES counties (geographic state subdivisions) have significantly higher CRC mortality than middle or high SES counties [5]. Rural-dwelling populations also have higher CRC incidence than urban populations across most of the U.S., with mortality rates falling at a slower rate [8]. Improved CRC screening rates for CRC is the leading factor in CRC mortality reductions [9]. However, lack of access to primary care providers (PCPs) and gastroenterologists (GIs) is associated with lower screening rates [10, 11].

In the U.S. state of Virginia, the CRC incidence and mortality rates (36.0 and 14.0 per 100,000, respectively) are slightly more favorable than national estimates (39.2 and 14.5 per 100,000, respectively) [12]. However, CRC county-level rates reveal wide variability, with incidence rates ranging from 22.7 to 70.0 per 100,000 and mortality rates from 8.2 to 32.5 per 100,000 [13]. Nationally, county-level incidence ranges 12.4 to 138.6 per 100,000 and mortality ranges 5.0 to 45.1 per 100,000 [12]. Statewide variations in the rates of CRC risk factors highlight the complexities of understanding their contributions to disease disparities. For example, the Appalachian southwest region of Virginia is very rural and has high poverty rates but has a low proportion of AA residents [14]. In contrast, the southeast region has higher densities of AA residents with a wide mix of rural-urban and SES status [14]. Previous studies have applied spatial analysis to understand how these underlying factors impact disparities and outcomes for prostate and breast cancer in Virginia [15, 16]. However, no known spatial analysis study has focused on CRC disparities in Virginia.

In addition to age-adjusted incidence and mortality rates, the mortality-incidence ratio (MIR) is a useful indicator possible cancer disparities. As a simple calculation of mortality rate divided by incidence rate from a simultaneous time period, a higher MIR indicates that more disease-related deaths occurred even if incidence remained steady [17]. MIR is not a direct measure of the likelihood that an individual diagnosed with cancer will survive [18]. However, differences in MIR across a population or region can indicate lack of healthcare access for cancer screening to find cancer in early stages, lack of facilities to treat cancer, or less effective survivorship care [19]. For example, in one study the amount of access to primary care through federally qualified health centers was found to significantly predict the MIR observed for breast, cervical, and prostate cancers, though non-significant results were found for CRC [20]. However, a multi-nation study found MIR for CRC to be predicted by healthcare access and quality [17].

Hotspot analysis is a method to identify regional disparities by detecting spatial clusters in which adjacent locations share high values, such as cancer incidence. Going beyond simply ranking high values by locality, hotspot analysis identifies regional patterns with multiple adjacent localities where disparities may be harder to overcome without traveling long distances. Hotspot analysis has been used internationally, such as a Brazilian study of CRC and an Iranian study of breast and prostate cancer [21, 22]. Examples from the U.S. include a U.S. national study to identify county clusters of high mortality rates [6] and a state-level study in Florida to identify areas with high rates of late-stage diagnoses [23].

Spatial regression and geographically weighted regression (GWR) provide a unique means of understanding associations between variables. In Tobler’s first law of geography, “everything is related to everything else, but near things are more related than distant things” [24]. In addition to identifying clusters, these spatial methods can also test for “non-stationarity” or the extent that relationships between variables, such as poverty and cancer incidence, vary geographically, though not necessarily by proximity [25]. Investigating spatial non-stationarity through analyses, such as GWR, can better identify the relative importance of each factor in a given region [26]. Spatial regression and GWR have been used to analyze disparities in CRC in international settings [27–29] and other cancer types in U.S. states and metropolises [15, 26, 30]; however, few such analyses have focused on CRC in the U.S.

The aim of this study is to assess CRC disparities in Virginia by (1) identifying locality-level clusters of high CRC incidence, mortality, and MIR and (2) describing spatial dynamics of demographic, socioeconomic, and healthcare access contributors to CRC disparities. This study can inform public health practitioners and healthcare administrators about populations and regions that could benefit from targeted CRC prevention, detection, and treatment programs.

## Methods

### Data sources and variables

Data at the county (*n* = 95) or city (*n* = 38) level were used in this study. Virginia’s independent cities have administrative boundaries separate from counties and are considered equivalent to counties in the U.S. Census [31]. The range in population for counties and cities is 2212 to 1,148,433 and 3936 to 450,435, respectively [32]. For the purposes of this study, the term “locality” is used to comprise both counties and independent cities.

Included in the analysis are three variables reflecting CRC outcomes: (1) incidence [12], (2) mortality [12], and (3) MIR [19]. Six independent variables included: (1) proportion of AA residents [32], (2) rural-urban status [33], (3) SES index [34], (4) CRC screening rate [35], (5) PCP density [36], and (6) GI density [36]. Table 1 provides definitions, data sources, and details for the variables. The most recent years of CRC data, 2011–2015, were used. Data for other variables were selected to overlap in timeframe with the CRC data when possible. However, for SES index the 2017 dataset was the closest available year [34], and CRC screening data were available only for 2008–2010 [35]. Data for independent variables were available for all 133 localities.

**Table 1:** Variable definitions, sources and measures and characteristics of the Virginia localities

Variable Name	Variable Type	Data Source; details	Measure	Mean [Minimum, Maximum]
Incidence	Outcome	State Cancer Profiles 2011–2015 [12]; annual estimate of invasive cancer diagnoses with 95% confidence interval. Age adjusted incidence based on 2000 US standard population.	Annual CRC diagnoses per 100,000 population	39.4 (22.7,70.0)
Mortality	Outcome	State Cancer Profiles 2011–2015 [12]; annual estimate of CRC attributable deaths with 95% confidence interval. Age adjusted incidence based on 2000 US standard population.	Annual CRC deaths, per 100,000 population	16.5 (8.2,32.5)
Mortality- incidence ratio (MIR)	Outcome	CRC mortality rate divided by incidence rate for each locality for which both data points were available	Ratio expressed as decimal	0.43 (0.26,1.08)
AA population	Independent	Racial category in US Census data 2010 [32]	Percent of population	18.9 (0.10,79.10)
Rural-urban status	Independent	US Department of Agriculture Economic Research Service 2013 [33]; Rural Urban Continuum Codes based on urban population within locality and adjacency to other metropolitan areas	Ordinal scores 1–9; 1–3 are metropolitan, 4–9 are non-metro	3.72 (1,9)
SES index	Independent	Appalachian Regional Commission 2017 [34]; composite index of unemployment, per capita income, poverty.	Index value based on 100 as US average; lower index indicates higher SES	101.6 (47.2166.7)
CRC screening rate	Independent	Small Area Estimates 2008–2010 [35]; estimates based on Behavioral Risk Factor Surveillance Survey question on self-reported previous screening.	Percent of population	63.1 (33.2,88.8)
PCP density	Independent	HRSA Area Health Resource Files 2011–2015 [36]; count for each year by locality	PCPs per 100,000 population; sum of PCPs for each year, divided by locality population for each year, then multiplied by 100,000	61.9 (0,323.2)
GI density	Independent	HRSA Area Health Resource Files 2011–2015 [36]; count for each year by locality	GIs per 100,000 population; sum of GIs for each year, divided by locality population for each year, then multiplied by 100,000	2.7 (0,43.3)

In all analyses, models were fit using complete case analysis, where localities with missing data (*n* = 6 for incidence, *n* = 30 for mortality, and *n* = 30 for MIR) for incidence, mortality, or MIR were excluded from their respective analyses. The cause of missing data was suppression of low counts for localities in the dataset, due to concerns for confidentiality and reliability [12].

### Getis-Ord GI* hotspot detection

A hotspot analysis was performed to identify spatial clustering of localities with high or low values for incidence, mortality, and MIR. These spatial clusters were calculated using the Getis-Ord GI* statistic (ArcGIS 10.4 software), which produces a Gi* statistic (z-score) and *p*-value assessing high (i.e., hotspot) or low (i.e., cold spot) spatial clustering, as described by Getis and Ord [37]. A pseudo-Plate Carrée linear map projection was used. Two parallel analyses compared results from the sample of Virginia localities only and a sample that included Virginia localities plus adjoining counties in bordering states. Since the results and interpretations were remarkably similar, only the results for the Virginia localities are presented here.

A False Discovery Rate (FDR) was applied to correct for multiple testing [38] to ensure that the rate of false positives in all tests chosen as significant would be at or lower than a set level. An FDR of 10% was used in the analysis. Although this criterion may not be as stringent as an FDR criterion of 5%, it is a more conservative approach than choosing the hotspots based on unadjusted *p*-values [39].

### Multivariate and spatial regression

Regression analyses were used to assess the contributions of the independent variables to the CRC outcomes. A linear regression was run as a preliminary step, to provide residuals for a test of spatial autocorrelation prior to spatial regression. The two spatial analyses, spatial regression and GWR, incorporated spatial autocorrelation into their models.

In the multivariate linear analyses, the three outcome variables (incidence, mortality, and MIR) were treated separately as a function of five predictors: proportion of AA residents, rural-urban status, SES index, CRC screening rate, and PCP density. The GI density variable was dropped from the analysis to avoid collinearity [40] because its high correlation (~ 0.80) with PCP density.

The residuals in the linear regression were then analyzed for spatial autocorrelation. Moran’s I and Geary’s C tests were performed to determine if spatial autocorrelation was present [41]. Clustering statistics suggested spatial autocorrelation was present for incidence (Geary’s C *p* = 0.0137, Moran’s I < 0.0001) and highly detectable for MIR (Geary’s c and Moran’s I *p* < 0.0001). As such, the regression model was extended to incorporate spatial autocorrelation. PROC MIXED in SAS was used to specify the covariance matrix, using the syntax described in Xu et al. [41], and using as geographical inputs the county centroid latitude/longitudes [42].

Next, GWR was used to assess for spatial non-stationarity between variables by testing whether the regression coefficients of the linear regression vary as a function of geography (i.e. β(x,y) as compared to β, where x,y are geo-coordinates) [43]. A semi-parametric GWR approach, in which the variables with locally varying coefficients were selected while holding other variable coefficients to be constant, was selected as offering the most parsimonious models and simplified interpretation. Locally varying coefficients were identified based on Akaike Information Criterion model fit. Maps display significant findings of local coefficients for each outcome variable (Fig. 3). Colors in the maps represent smoothed values of the non-stationary parameters presented over the geographical region. Red indicates a more positive relationship between the independent and outcome variable, while blue indicates a more negative relationship. In all cases, these parameters were geographically varying regression weights illustrating the local level of association between predictor and outcome.

## Results

**Fig. 1:**
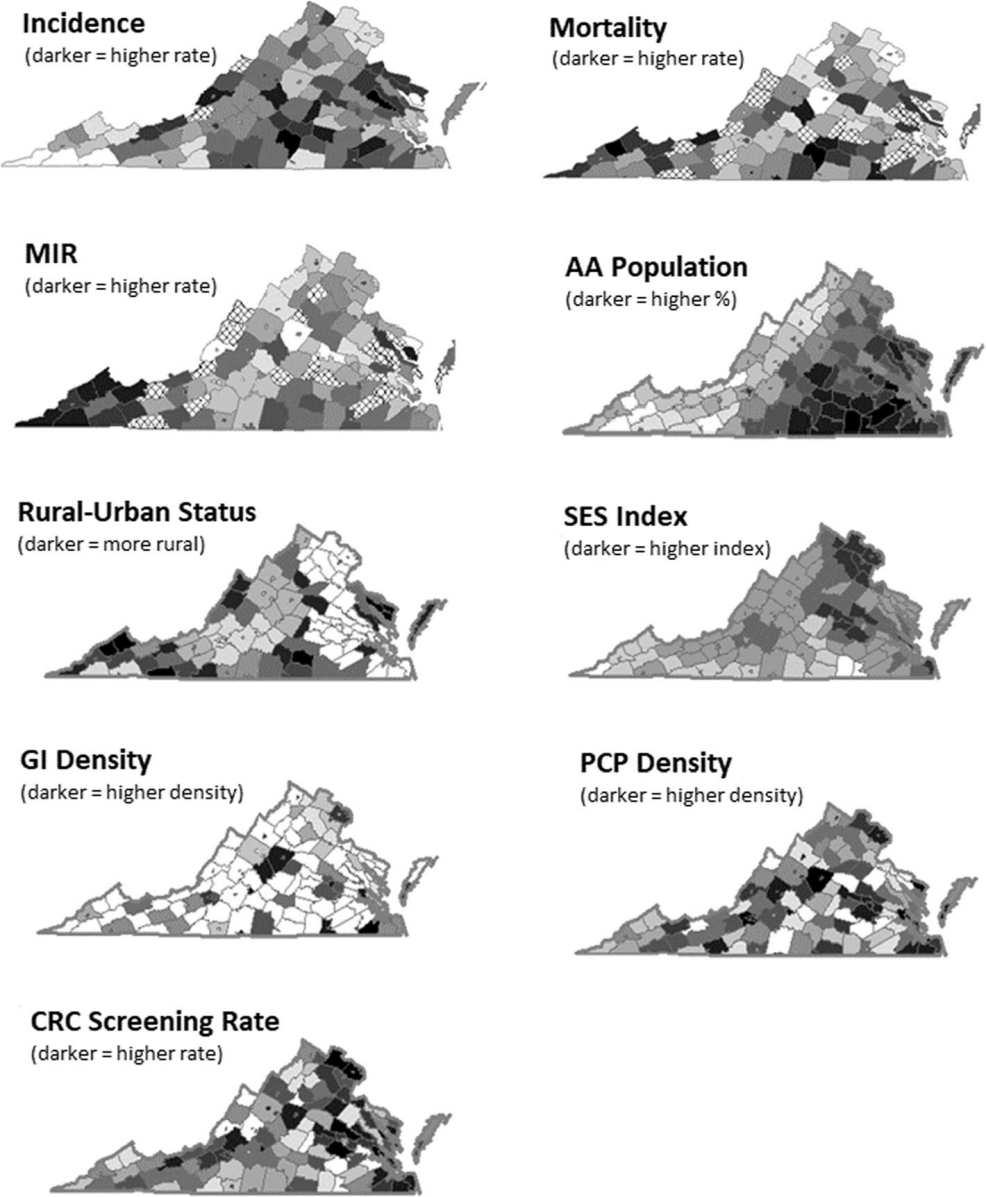
Distribution of independent variables across Virginia counties and cities; choropleth maps with values displayed as quantiles. Crosshatch marks in Incidence, Mortality, and MIR indicate areas with missing data. Map created by the authors, using ArcGIS 10.4 licensed to University of Virginia

The statewide distributions of each variable are presented as choropleth displays in Fig. 1. In visual estimation, incidence had higher rates in the central and eastern regions, and pockets of low rates in the northern and southwest regions. Patterns of high mortality were observed in the southwest and south-central regions. For MIR, a high rate was concentrated in the southwest region. The proportion of AA population was highest in the south-central and southeast portions of the state, and lowest in the southwestern areas. With major cities in the northern and eastern parts of the state, widespread rurality was more pronounced in the southwest and south-central regions, as well as near the eastern seaboard. SES index tended to be lowest in southwest and south-central regions, and highest in northern Virginia. PCP density and CRC screening rates had varying values but without a visible pattern of distribution.

**Fig. 2:**
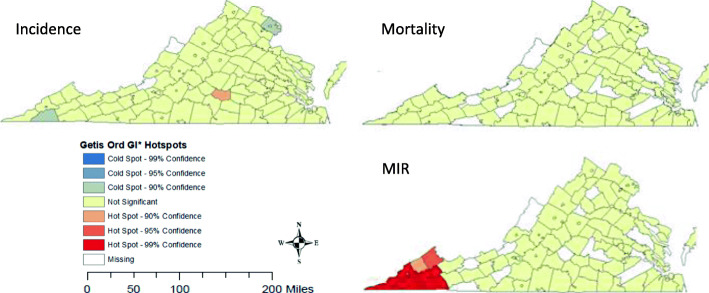
Getis-Ord GI* hotspot maps of CRC incidence, mortality, and mortality-incidence ratio (MIR). Map created by the authors, using ArcGIS 10.4 licensed to University of Virginia

Figure 2 displays hot and cold spots for each of the outcome variables. For incidence, a hotspot was detected in the rural south-central part of the state (*p* < 0.10). Two cold spots were detected, in the far southwest corner (*p* < 0.10) and in the northern area near Washington DC (*p* < 0.10). For mortality, no hotspots or cold spots were detected. The MIR detected a single, large hotspot encompassing the southwest corner (*p* < 0.01).

Table 2 shows the regression parameter estimates between the independent variables and the three CRC outcome variables. Adjusting for all predictors, all three overall models were significantly predictive (*p* < 0.001). In relation to incidence, the only significant single predictor was proportion of AA residents (*p* < 0.0001), such that an increased proportion of AA coincided with increased CRC incidence. For mortality, rural-urban status was a significant predictor (*p* = 0.018), indicating that increased rurality was associated with increased mortality. SES index was also significantly associated (*p* = 0.004), with lower SES index associated with higher mortality. For MIR, the only significant variable in the model was SES index (*p* = 0.004), indicating that lower SES index was associated with higher MIR.

**Table 2:** Multivariate and spatially correlated regression model estimates for CRC incidence, mortality, and MIR

	Linear Model with independent errors				Spatially Correlated Model			
	Beta	Std Err	Std Beta	*p*-value	Beta	Std Err	Std Beta	*p*-value
**Incidence**								
Intercept	35.87	8.24	39.3	<.000	28.90	10.32	35.38	0.008
Rural-urban status	0.560	0.334	1.57	0.094	0.700	0.289	1.95	0.018
CRC screening rate	−0.046	0.109	−0.35	0.675	0.020	0.113	0.16	0.859
AA population	0.206	0.049	3.45	<.001	0.230	0.073	3.86	0.002
PCP density	−0.008	0.013	−0.42	0.558	− 0.015	0.015	− 0.83	0.310
SES index	0.008	0.031	0.24	0.790	−0.008	0.048	−0.23	0.869
Overall Test p-val	< 0.001				< 0.001			
**Mortality**								
Intercept	10.91	5.184	16.63	0.035	10.91	5.34	16.63	0.044
Rural-urban status	0.398	0.168	1.12	0.018	0.398	0.173	1.12	0.024
CRC screening rate	−0.013	0.064	−0.10	0.834	−0.013	0.068	−0.10	0.842
AA population	0.047	0.025	0.79	0.063	0.047	0.024	0.79	0.050
PCP density	−0.013	0.008	−0.71	0.089	−0.013	0.008	−0.71	0.119
SES index	0.049	0.017	1.44	0.004	0.049	0.018	1.44	0.007
Overall Test p-val	< 0.001				< 0.001			
**MIR**								
Intercept	0.068	0.141	0.43	0.633	0.329	0.181	0.51	0.085
Rural-urban status	0.006	0.007	0.02	0.360	0.008	0.004	0.02	0.085
CRC screening rate	0.003	0.002	0.02	0.126	0.002	0.002	0.01	0.332
AA population	−0.001	0.001	−0.02	0.129	0.001	0.001	0.02	0.208
PCP density	−0.0002	0.0003	−0.01	0.357	−0.0001	0.0002	−0.01	0.566
SES index	0.002	0.001	0.05	0.004	0.0003	0.001	0.01	0.085
Overall Test p-val	< 0.001				0.142			

*Std Err* Standard error, *Std Beta* Standardized beta coefficient

For the spatially correlated regression (Table 2), the overall models for incidence and mortality were significant (*p* < 0.001), while the MIR model was not (*p* = 0.142). For incidence, an increased proportion of AA population (*p* = 0.002) and increased rurality status (*p* = 0.018) had significant positive associations. In terms of mortality, increased rurality status (*p* = 0.024) and lower SES index (*p* = 0.007) had significant positive associations.

**Fig. 3:**
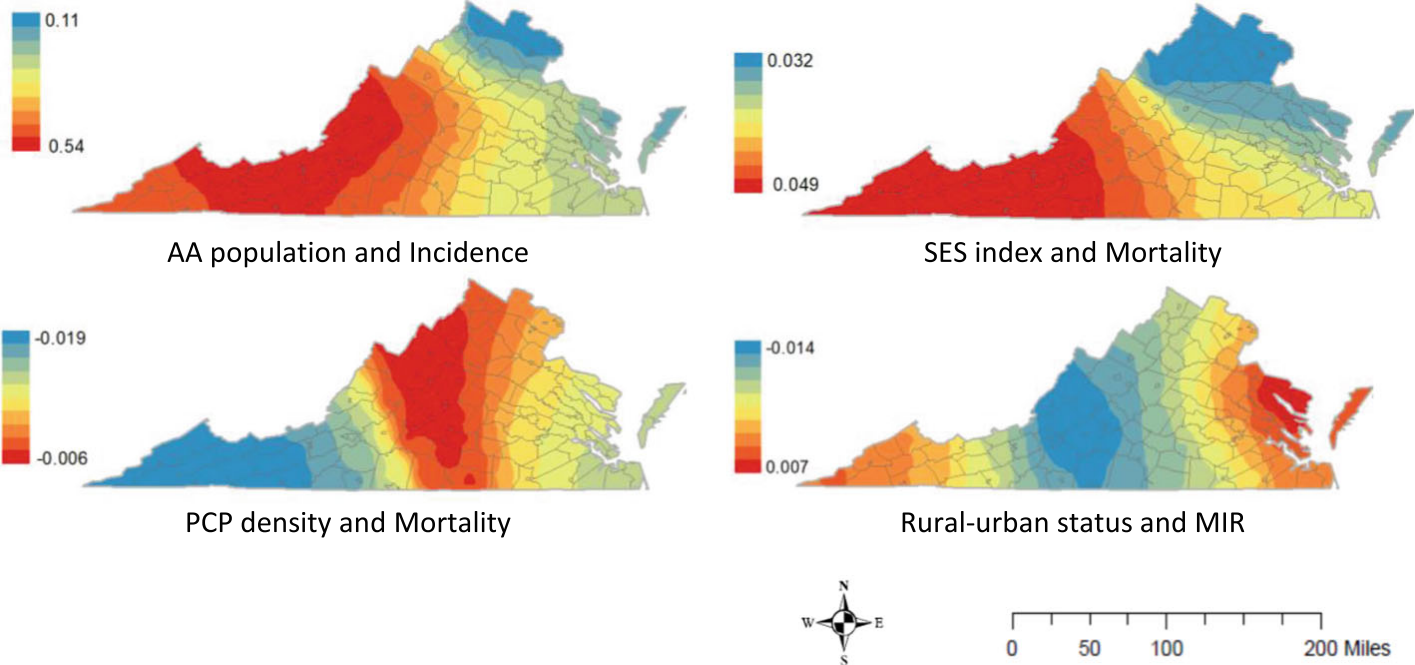
Geographically weighted regression local coefficients. Red indicates more positive relationship between independent and outcome variables; blue indicates more negative relationship. Map created by the authors, using ArcGIS 10.4 licensed to University of Virginia

Under the GWR model, non-stationarity was observed in the varying strength of association between variables. The association between percentage of AA population and incidence was found to vary geographically but remain positive (GWR local betas from 0.14–0.52, median = 0.24), with a stronger association in southwestern Virginia compared to the rest of the state (Fig. 3). The association of SES index with mortality was found to vary geographically and in the same direction (GWR local betas from 0.032 to 0.049, median = 0.034) such that SES was a stronger predictor of mortality in southwestern Virginia, than other regions. Similarly, the association of PCP density with mortality was found to vary geographically in the same direction of increasing PCP supply with decreasing mortality (GWR local betas from − 0.019 to − 0.006, median = − 0.009). The effect of PCP supply on mortality was strongest in southwestern Virginia and weakest in central Virginia. For rural-urban status, the GWR model detected geographical variation in its association with MIR in varying directions (local betas from − 0.014 to 0.007, median − 0.002) with positive associations in the eastern seaboard and southwest, and negative associations in central Virginia, suggesting that increasing rurality was associated with increasing MIR in the eastern and southwestern regions of the state but decreasing MIR in the central part.

Summarizing results for incidence, the hotspot in south-central Virginia represents a rural and lower SES region with a high proportion of AA population relative to other regions. The cold spots for incidence were found in two widely contrasting regions including southwest Virginia as rural with high poverty and mostly white residents, and the northern region as urban, more racially diverse and one of the wealthiest parts of the state [14]. Of the independent variables, increasing rurality and proportion of AA residents were positively associated with incidence. Rural-urban status association was significant in the spatial regression, while the proportion of AA residents was significant in the linear and spatial regression model. The GWR model for CRC incidence showed a stronger association for the proportion of AA residents in the western part of the state.

For mortality, no significant hotspots were identified. However, relative to the state average, locality-level mortality rates were generally higher in the southwest region and lower in the northern region. Lower SES index was associated with increased mortality in the linear and spatial regressions. The GWR indicated non-stationarity of this association, showing a stronger association between SES index and mortality in southwestern VA. Rurality was also associated with increased mortality in the linear and spatial regression models. PCP density in association with mortality was found to be non-stationary in the GWR local coefficient, indicating that an inverse association was stronger in southwestern Virginia and weaker in the central regions.

For MIR, a large and strongly significant hotspot was found in southwestern Virginia. The GWR found the association of rurality and MIR to be non-stationary, with stronger positive associations in the southwestern and eastern edges of the state, and slightly negative associations in the central regions. Low SES was associated with higher MIR in the linear but not the spatial regression.

## Discussion

This study found disparities in CRC outcomes in the southwest and south-central regions of Virginia, and that key factors of disparities are rurality, low SES index, and high proportion of AA population. An important finding from our study is that the strength of the association of SES index, race, and PCP density with both incidence and mortality varies across regions of the state. Likewise, rurality was linked to both increased and decreased mortality depending on region. Collectively, findings indicated that many risk factors for CRC incidence or mortality may gain or lose strength on a regional basis, perhaps by combining with other factors or even as a result of community-level interventions. Future studies could assess CRC prevention and control services in relation to risk factors and outcomes.

The MIR hotspot in southwestern Virginia was concerning in terms of potential widespread disparities in access to the continuum of cancer care from detection through treatment. The finding of a cluster of low CRC incidence (a cold spot) in an overlapping location was unexpected and does not reflect other studies finding incidence disparities in rural and especially in Appalachian regions. Previous studies of cancer disparities comparing Appalachian and non-Appalachian populations have found higher overall cancer incidence in Appalachian populations [44, 45] as well as higher CRC incidence [44]. However, a Virginia state cancer report found southwestern health districts had relatively low incidence but high mortality as well as poor rates of detecting cancer at an early stage [13]. Low incidence with high mortality, as denoted by the MIR, could indicate lack of early diagnosis and under-diagnosis, possibly due to low CRC screening rates. Additional assessment is warranted to better understand the dynamics of low incidence rates and high MIR in southwest Virginia. The use of spatial hotspot analysis bolstered evidence that regions with concentrations of social or economic disadvantage may face additional hurdles to improving CRC outcomes. A national CRC mortality study found hotspots occurred in regions characterized by poverty, rurality, and/or high proportion of AA residents [6]. The current study added depth to the understanding of these geographic disparities through state-level analysis of incidence, mortality, and MIR.

The associations of population characteristics with CRC outcomes reinforced previous studies. Rurality and low SES index were predominant factors in CRC mortality. Both of these population types are characterized by social and structural barriers to cancer prevention and accessing healthcare services along the cancer control continuum [46–48]. The finding of higher incidence among populations with greater proportions of AA residents bears further assessment of local risk factors. However, this finding is also reflective of larger trends of obstacles to healthcare access among AA populations such as lower average SES status than white populations and higher levels of distrust in healthcare organizations [49–51].

The more proximal factors of health care access, PCP density and CRC screening rates, offered additional insights. The finding that PCP density had a stronger negative association with mortality in southwest Virginia compared to the central region is consistent with other studies noting the important role of PCPs in cancer screening and care access, particularly in rural areas where specialists are less accessible [20, 52, 53]. It is notable that CRC screening rate itself was not associated with any of the CRC outcome variables. Previous studies have found strong evidence that screening rates are associated with lower rates of both incidence and mortality and that implementing screening programs results in reductions in these outcomes [44, 54, 55]. Rurality has also been associated with lower screening rates in a national study [56]. The Behavioral Risk Factor Surveillance Survey (BRFSS), the source for screening rates in the current study, might also be a factor. A past study found BRFSS responses overestimated CRC screening rates compared to other surveys [57], and another Virginia-based study using BRFSS prostate cancer screening data also found no association with prostate cancer incidence [16].

The non-stationarity results in this study indicate that a “one-size-fits-all” approach may not be ideal due to variations in the strength of association between population characteristics and CRC outcomes. Rather, these results provide new insight on local or regional solutions that could help improve cancer outcomes in high-disparity parts of the state (Fig. 3). For example, increased access to PCPs in the southwestern region could have a larger effect on CRC mortality. Similarly, the results suggest that programs to improve low SES individuals’ access to early detection and treatment could have greater impact on mortality in the southwestern region. Non-stationarity analysis can also shed light on geographic areas and population characteristics for whom targeted cancer interventions might have greater impact. A previous study found that strength of the association of area-based economic deprivation and later stage breast tumors was place-specific and was stronger in the Appalachian regions of Pennsylvania than in either Ohio or Kentucky [30]. Other non-stationarity studies include an analysis of U.S. prostate cancer mortality that found urologist availability had stronger association in certain regions [58], and a English study that found regions with stronger associations between low SES and cervical cancer incidence [59]. Identifying associations that have spatial variability is important for selecting data analysis approaches that yield more precise results.

There are limitations in this study. The use of ecological data rather than individual-level data increases the likelihood of erroneous findings, due to aggregations of population data that do not accurately reflect the characteristics or travel patterns of individuals in those localities. Yet, as all the data used in this study were publicly available, this study provides an example for population health programs wishing to conduct spatial analyses but unable to access individual-level data. Available data for CRC screening estimates were older than incidence and mortality data, causing potential errors in analyzing associations. The missing data, particularly for mortality and MIR, may have caused errors in analyses.

### Research and practice implications

The spatial methods used in this study can be replicated in other geographic settings to elucidate some of the complex factors of CRC outcomes. Future spatial analysis using individual-level, rather than locality-level, CRC data can add to understanding of statewide populations and regions in Virginia at risk for cancer disparities. Future analyses should also include variables more proximal to cancer outcomes, such as late-stage diagnosis, statewide availability and accessibility of cancer screening, and specialist treatment resources. Further research is needed to understand the low incidence rates in the otherwise health disparate southwest Virginia region, especially whether this is due to under-detection, and to explore the lack of association between CRC screening rates and outcomes.

Practitioners can use this study’s findings to target programs and policies to specific regions and adapt interventions to better meet the needs of high-disparity population groups. For example, programs and policies to lower cost barriers to CRC screening and treatment should be targeted to low SES populations. Programs for CRC prevention and early screening should be tailored and targeted to effectively reach AA populations. Similarly, outreach programs should be tested and adapted for implementation in rural communities [60]. For areas with high MIR, programs and policies should target increased access to PCPs, screening, early detection, and CRC treatment specialists.

## Conclusions

This study of the U.S. state of Virginia found that rurality, low SES index, proportion of AA population, and low density of PCPs were associated with CRC outcome disparities. The strength of associations varied spatially, indicating addressing risk factors in areas with stronger associations may yield greater impact on CRC outcomes. Spatial analyses such as hotspot clusters and non-stationarity are important for understanding nuanced attributes of risk factors for CRC.

## Data Availability

Data used in this analysis are available for open public access at: State Cancer Profiles. Quick profiles: Virginia. 2019; Available from: www.statecancerprofiles.cancer.gov. University of Wisconsin Population Health Institute, *County Health Rankings & Roadmaps 2019*, https://www.countyhealthrankings.org/ United Stated Department of Agriculture Economic Research Service. *Rural-urban continuum codes*, https://www.ers.usda.gov/data-products/rural-urban-continuum-codes/. Appalachian Regional Commission. *County economic status in Appalachia, FY 2017*, https://www.arc.gov/research/MapsofAppalachia.asp. National Cancer Institute, *Small area estimates for cancer-related measures: CRC test ever,* https://sae.cancer.gov/nhis-brfss/estimates/crc.html Health Resources & Services Administration, *Area health resources files*, https://data.hrsa.gov/topics/health-workforce/ahrf

## Abbreviations

AA: African American
CRC: Colorectal cancer
FDR: False discovery rate
GI: Gastroenterologist
GWR: Geographically weighted regression
MIR: Mortality-incidence ratio
PCP: Primary care provider
SES: Socioeconomic status
U.S.: United States
